# Preparation of Composites Derived from Modified Loess/Chitosan and Its Adsorption Performance for Methyl Orange

**DOI:** 10.3390/molecules29215052

**Published:** 2024-10-25

**Authors:** Haobin Hu, Haiyan Song, Zhenyu Cheng, Yufeng Wang, Qi Zhang, Huaisheng Hu, Lala Zhang

**Affiliations:** Gansu Key Laboratory of Efficient Utilization of Oil and Gas Resources in Longdong, Longdong University, Qingyang 745000, China; haiyan514319614@163.com (H.S.); chengzhenyu0633@126.com (Z.C.); ldxyhhs@163.com (H.H.); lalazhang@126.com (L.Z.)

**Keywords:** modified loess, chitosan, composites, methyl orange, adsorption kinetics, adsorption isotherm

## Abstract

A modified loess/chitosan composite (ML@CS) was prepared via solution. The microstructure and physicochemical properties of ML@CS were characterised via scanning electron microscope (SEM), Zeta potential, X-ray diffraction (XRD), Fourier transform infrared spectrum (FT-IR), and thermogravimetric analysis (TGA). An aqueous solution of methyl orange (MO) was used as simulated wastewater from which the influence of the initial concentration and pH of MO, the dosage amount and regeneration performance of ML@CS, adsorption temperature, and time on the adsorption effect of MO were systematically investigated. The adsorption kinetics, isothermal adsorption, and adsorption mechanism were also analysed. The results indicate that ML@CS had a good adsorption effect on MO. When the initial concentration of MO was 200 mg/L, pH was 5.0, and ML@CS dosage was 1.0 g/L, the adsorption equilibrium could be reached within 180 min at room temperature, and the equilibrium adsorption capacity and removal rate reached 199.52 mg/g and 99.75%, respectively. After five adsorption–desorption cycles, the MO removal rate remained above 82%. The adsorption behaviour of ML@CS for MO conforms to the pseudo–second–order kinetic model and the Langmuir isotherm adsorption model. The spontaneous exothermic process was mainly controlled by monolayer chemical adsorption and the physical adsorption only played an auxiliary role. ML@CS efficiently adsorbed MO in water and can be used as a high-efficiency, low-cost adsorbent for printing and dyeing wastewater treatment.

## 1. Introduction

The amount of wastewater generated by the printing and dyeing industry, owing to its rapid development, has been increasing dramatically. This wastewater has high chemical oxygen demand (COD) and chromaticity, complex components, high biological toxicity, difficult natural degradation, and requires treatment [1]. In particular, azo dyes or methyl orange (MO) have potential carcinogenic, teratogenic, and mutagenic effects on organisms and are one of the refractory industrial wastewaters [2]. Among the various technologies used for azo dye wastewater treatment, adsorption is effective and commonly used [3]. However, most of the traditional adsorbent materials (such as activated carbon, resin, molecular sieve, inorganic and organic polymer flocculant, etc.) have some evident shortcomings such as difficult separation, easy loss, poor recycling, serious secondary pollution, and high preparation cost [4,5]. Therefore, the development of environment-friendly adsorption materials with high efficiency, economic viability, and good recycling performance is one of the hot spots in the field of wastewater treatment research.

Chitosan (CS) is a linear polymer with a high molecular weight. It is obtained by the partial deacetylation of the natural polysaccharide chitin, which contains active groups such as hydroxyl (–OH), amino (–NH_2_), glycosides, and *N*-acetyl amino groups in its molecular chain [5,6]. Owing to the formation of a secondary structure from the hydrogen bonds between the skeleton chain structures, CS exhibits the following effects: ion exchange, chelation, adsorption, crosslinking, flocculation, biocompatibility, and degradation of most organic compounds and metal ions [7,8,9,10]. However, the applicability of CS for industry is limited, to some extent, by virtue of its high crystallinity, low porosity and mechanical strength, weak resistance to acid, poor chemical stability, difficult solid-liquid separation and recovery, and easy-to-cause secondary pollution [7]. Owing to its typical high porosity, weak cementation and metastable structure with loose filling, and active points, including silicon and aluminium, as well as good dispersibility, large specific surface area, and strong chemical resistance, loess exhibits excellent adsorption and cementation properties [11,12], and is applied in the field of fine chemical industry [13,14,15]. However, the functional groups on the surface of loess are few, the hydrophilicity is strong, the adsorption capacity is limited, and the selectivity is poor, which limits its practical application in wastewater treatment.

To exploit the synergistic effect of loess and chitosan in printing and dyeing wastewater treatment, improve the physical and chemical stability, and enhance the mechanical strength and regeneration performance of the adsorption material, a composite was prepared by modifying loess to have the characteristics of a resource-rich, low-cost, non-toxic, harmless, and environmentally friendly material. CS was loaded onto the modified loess (ML) and its adsorption property was explored. This study provides new ideas for the development and utilisation of green, efficient, and low-cost materials for the treatment of wastewater containing MO.

## 2. Results and Discussion

### 2.1. Modified Loess

Firstly, water-soluble impurities, suspended solids, and sand particles in loess were removed by washing with water, and then the acid-soluble substances in loess were removed by acidification using hydrochloric acid (HCl). This process reduced the ash content in the loess and enlarged its specific surface area, thereby improving its adsorption performance. More importantly, the exchange reaction between H^+^ and cations in the loess released more silicon hydroxyl group (Si–OH) from the surface of loess particles, which was favourable for the combination of loess and CS [16].

### 2.2. Material Characterisation

#### 2.2.1. Appearance and Morphology

**Figure 1:**
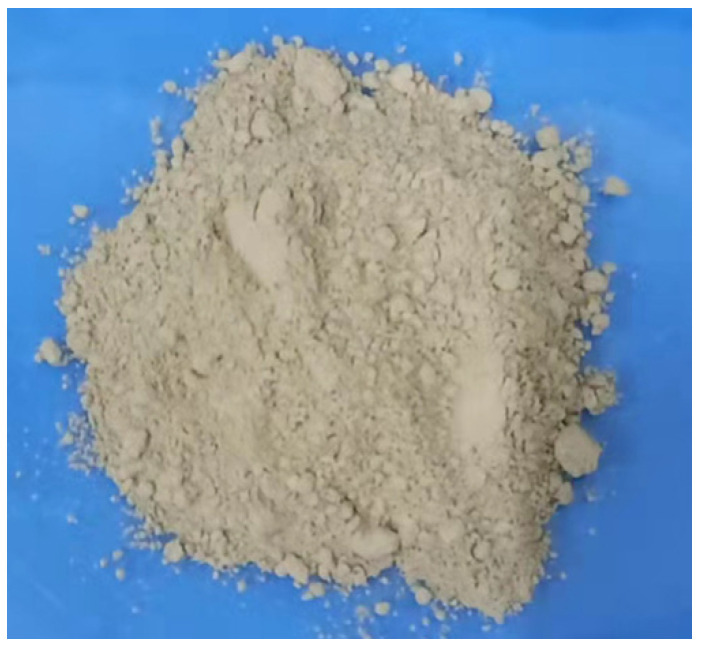
Appearance of ML@CS.

**Figure 2:**
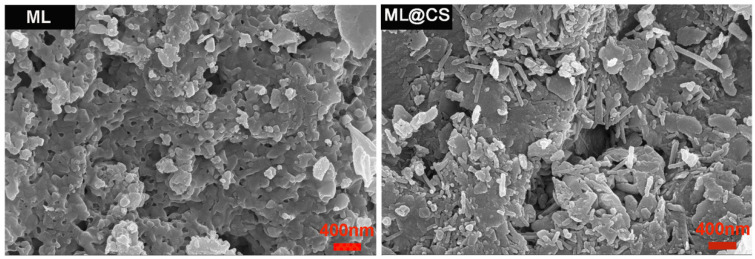
SEM images of ML and ML@CS.

CS was loaded onto the surface of the modified loess, which yielded a light-yellow powdered ML@CS composite (Figure 1). The morphological characteristics of ML and ML@CS were explored via SEM. The results are shown in Figure 2. The BET data of ML and ML@CS are shown in Table 1.

A comparison of the SEM images of ML and ML@CS shows layered structures with irregular textures stacked together and unevenly distributed pores on the surface of ML particles, whereas the agglomerates of ML@CS appear to be more dispersed, and a large number of irregular particles are attached to the surface of the agglomerates. Notably, the layered structure of the loess in ML@CS has not completely disappeared, indicating that loess and CS have been successfully compounded. Furthermore, it can be seen from Table 1 that although there is little difference between the average pore volume and average pore size of ML@CS and ML, the specific surface area of ML@CS is 48.71 m^2^/g larger than that of ML, indicating that CS loading on the surface of ML not only increases the specific surface area and adsorption site of ML, but also acts as a bridge to bind the loess particles. At the same time, the introduction of CS hinders the stacking between ML particles, which was conducive to improving the adsorption performance.

#### 2.2.2. Zeta Potential Analysis

**Figure 3:**
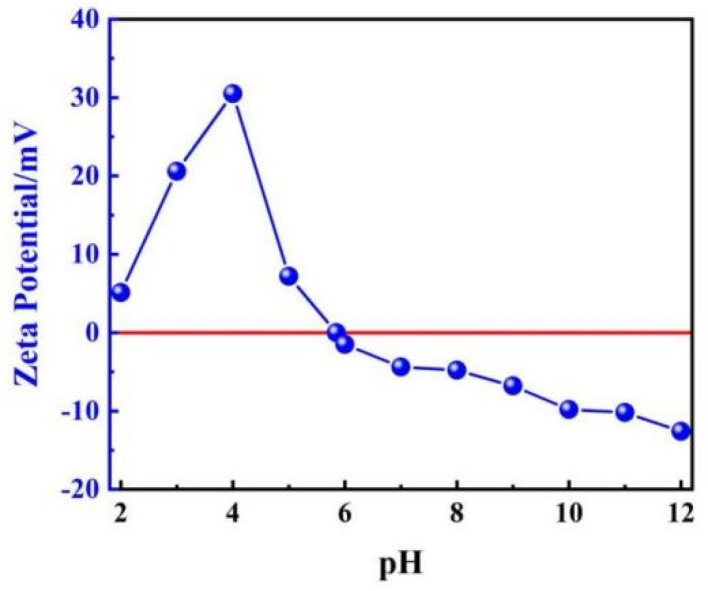
Effect of pH on Zeta potentials of ML@CS. The red line means the Zeta potential is 0.

The Zeta potential of ML@CS as a function of pH is shown in Figure 3.

The surface electrical properties of the adsorption materials influence their adsorption properties. The adsorption efficiency and capacity can be substantially improved by selecting the adsorbate based on its surface charge properties and the size of the adsorbent. Figure 3 shows that the Zeta potential is positive when the pH is between 2 and 5, indicating that the ML@CS surface is positively charged. When pH > 6, the Zeta potential is negative and the isoelectric point is 5.85.

#### 2.2.3. XRD Analysis

**Figure 4:**
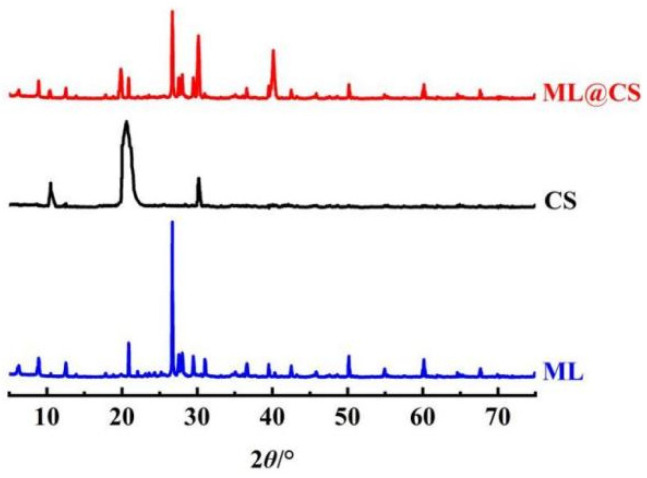
XRD patterns of the samples.

By comparing the XRD patterns in Figure 4, it is found that ML@CS shows the typical diffraction peaks of loess at 2*θ* = 22.1°, 28.0°, 31.0°, and 36.6°, indicating that the main composition of loess does not change during the preparation of ML@CS [17]. Moreover, the characteristic diffraction peaks of CS appear at 2*θ* = 10.5°, 20.6°, and 30.2° [18], a new diffraction peak appears at about 2*θ* = 40.1°, and the wider amorphous structure peak (2*θ* = 19.8°) in CS is shifted, indicating that CS has successfully combined with ML. In addition, the amorphous region of CS is enlarged and the crystallinity is reduced [19], but the basic structure of CS is not damaged, and there is still a certain amount of –OH and –NH_2_ on the surface that can adsorb MO well [20].

#### 2.2.4. FT-IR Analysis

**Figure 5:**
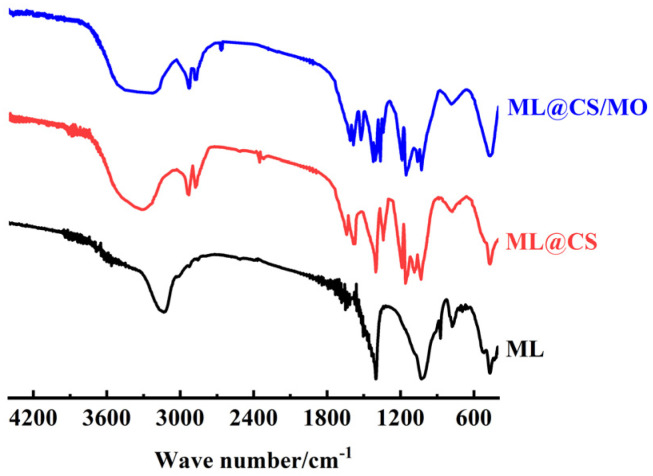
FT-IR spectra of the samples.

As can be seen from Figure 5, the peaks at 471 cm^−1^ and 778 cm^−1^ are assigned to the Si–O–Si bending vibration and the Si–O stretching vibration absorption, respectively. A strong absorption peak at 1086 cm^−1^ is formed by the superposition of the Si–O–Si stretching vibration absorption and the C–O stretching vibration absorption. The peaks at 2930 cm^−1^ and 2874 cm^−1^ can be ascribed to the stretching vibration absorption of C–H in –CH_3_ and –CH_2_– [21], and those at 1630 cm^−1^ and 1402 cm^−1^ can be attributed to the O–H and C–H bending vibration absorption, respectively. The peak at 1186 cm^−1^ is ascribed to the C–N stretching vibration absorption, and the weak amide absorption peak can be observed at 1341 cm^−1^. The peak around 1159 cm^−1^ can be ascribed to the C–O stretching vibration absorption, and those peaks around 1080 cm^−1^ and 1032 cm^−1^ belongs to the C_3_–OH and C_6_–OH stretching vibrations absorption in CS, respectively [22]. The peaks at 1572 and 1639 cm^−1^ are assigned to the in-plane bending vibration absorption of N–H in –NHCO– and the stretching vibration absorption of C=O, respectively [23].

The absorption peak at 1402 cm^−1^ belongs to the –COOH stretching vibration, which can be attributed to the natural organic matter composition in loess [24,25]. The multiple broad absorption peak at ~3200–3450 cm^−1^ originated from the overlap and broadening of the absorption peaks of O–H and N–H stretching vibrations, indicating the presence of intramolecular and intermolecular hydrogen bonds between –OH and –NH_2_ in CS molecules [26]. A strong –NH_2_ shear vibration absorption peak was observed at 1585 cm^−1^, indicating that –NH_2_ on CS is not destroyed during the ML@CS synthesis. These results confirm the successful synthesis of ML@CS by loading CS on the ML surface.

After the adsorption of MO on ML@CS, the mixed absorption peaks of N–H and O–H at 3415 cm^−1^ shifted to 3222 cm^−1^ and the peak intensity decreased, demonstrating that the groups originating from MO may form hydrogen bonds with –OH and –NH_2_ on ML@CS. At the same time, the bending vibration and skeleton vibration absorption peaks of benzene appeared at 1421 and 1610 cm^−1^, respectively. Similarly, the N=N vibration absorption peak was observed at 1521 cm^−1^, the C−N stretching vibration absorption peaks appeared at 1153 cm^−1^ and 1027 cm^−1^ and the S=O stretching vibration absorption peak appeared at 1364 cm^−1^. These characteristic peaks manifested the existence of a basic skeleton of MO and RSO_3_^−^ [27]. This confirms that an interaction occurred between –OH and –NH_2_ in ML@CS and the groups in MO [28,29]. Thus, –OH or –NH_2_ on the molecule must be protected during ML@CS synthesis.

#### 2.2.5. TGA

**Figure 6:**
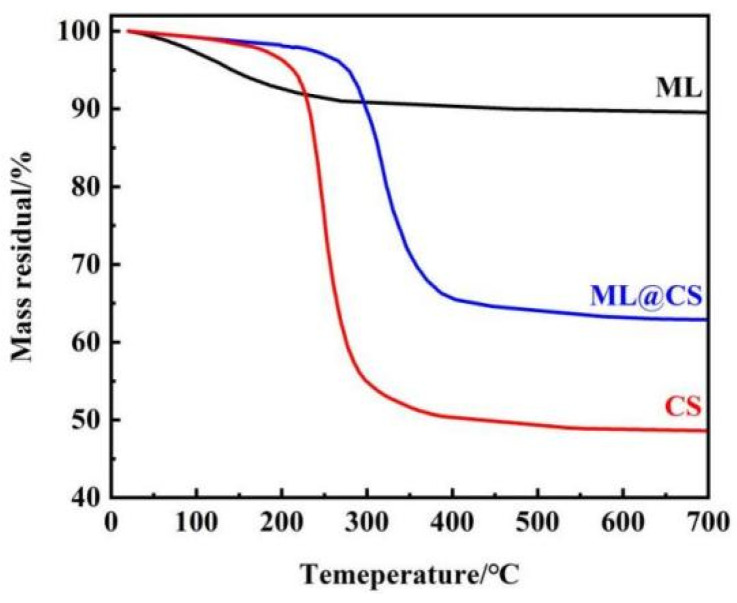
TGA curves of the samples.

Figure 6 shows the TGA curves of ML, CS, and ML@CS.

At room temperature to 250 °C, the weight loss rate of ML is 8.59%, which is mainly caused by the evaporation of adsorbed water in the loess surface and void. At 250–700 °C, the weight loss rate of ML is 1.84%, mainly resulting from the partial loss of bound water and decomposition of organic matter. The weight loss of CS is 0.78% due to the loss of water molecules when the temperature increases from room temperature to 100 °C. At 100–400 °C, the rapid weight loss of CS is 48.87% because of the loss of strong binding water and the degradation of CS [30]. As the temperature increases from room temperature to 200 °C, the weight loss of ML@CS is 1.91% due to the loss of a small amount of water molecules. At 200–450 °C, the weight loss of ML@CS is 33.53% because of the oxidative decomposition of CS. As the temperature increases from room temperature to 700 °C, the total weight loss rate of ML@CS is 37.09%, which is higher than that of ML and lower than that of CS. The weight loss of ML@CS is relatively slow, confirming the formation of a composite using CS and ML, ML@CS also has good thermal stability.

### 2.3. Influence of Adsorption Conditions on the Adsorption Effect

#### 2.3.1. Effect of Initial Concentration

**Figure 7:**
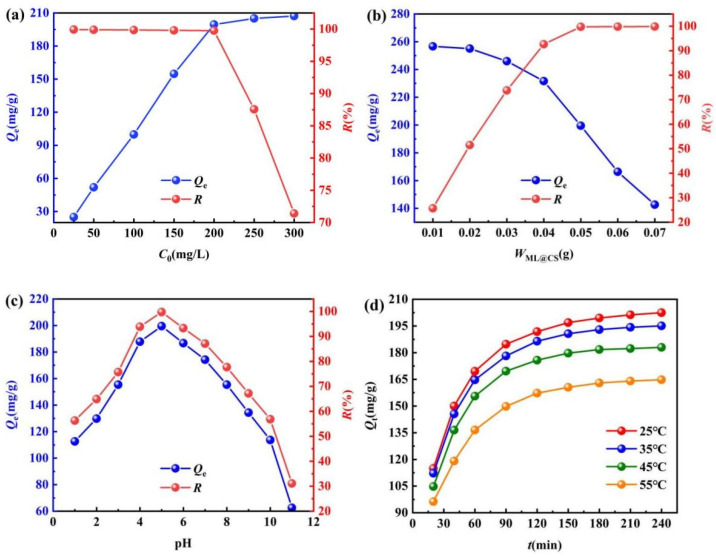
Effects of various factors on MO adsorption. (**a**) Effect of initial concentration at 0.05 g W_ML@CS_, pH 5.0, 25 °C and 180 min, respectively. (**b**) Effect of W_ML@CS_ at pH 5.0, 200 mg/L, 25 °C and 180 min, respectively. (**c**) Effect of pH at 0.05 g W_ML@CS_, 200 mg/L, 25 °C and 180 min, respectively. (**d**) Effect of adsorption time and adsorption temperature at pH 5.0, 0.05 g W_ML@CS_, 200 mg/L, respectively.

The effects of the initial concentrations (25, 50, 100, 150, 200, 250, and 300 mg/L) on the adsorption effect of ML@CS for MO were investigated at 25 °C, W_ML@CS_ of 0.05 g, pH of 5.0, and adsorption time of 180 min. As shown in Figure 7a, *Q*_e_ increases rapidly from 24.98 to 199.53 mg/g with a continuous increase in *C*_0_ from 25 to 200 mg/L for a constant ML@CS amount, whereas *R* gradually decreases from 99.92% to 99.75%. The residual concentration of MO in the water after treatment is< 0.5000 mg/L, which meets the emission standards. *Q*_e_ gradually increases to 207.20 mg/g when *C*_0_ is increased to 300 mg/L, whereas *R* considerably decreases to 71.41% because the adsorption sites of ML@CS did not reach a saturation state at low concentrations. The driving force for mass transfer of the solution increases with *C*_0_, and more amounts of MO are adsorbed on the ML@CS surface, resulting in a rapid increase in *Q*_e_ [31] and a gradual decrease in *R*. When the adsorption sites on the ML@CS surface are almost occupied completely, the excess MO can only be in a free state in the solution. Thus, *Q*_e_ increases gradually and *R* decreases considerably. Therefore, the optimal initial concentration of MO was regarded as 200 mg/L.

#### 2.3.2. Effect of the Addition Amount of ML@CS

The adsorbent amount is an important index for investigating the adsorption effect. When the dosage was insufficient, the adsorption effect was poor. Moreover, the pollutant could not be completely removed and the polluted water was not well treated. However, excessive amounts will decrease the adsorption efficiency and result in resource wastage and increase the cost. Thus, adding an appropriate amount of adsorbent will yield excellent treatment effect for polluted water and be economical. The effect of ML@CS dosage on MO adsorption was exploited when *C*_0_ was 200 mg/L, pH was 5.0 and adsorption temperature and time were 25 °C and 180 min, respectively. As shown in Figure 7b, *R* increases considerably at first and then increases gradually, whereas *Q*_e_ first decreases gradually and then substantially after the addition of ML@CS. After the ML@CS amount is increased from 0.01 to 0.05 g, *R* increases considerably from 25.67% to 99.76% and *Q*_e_ decreases gradually from 256.65 to 199.52 mg/g. When the ML@CS amount is further increased to 0.07 g, *R* increases gradually to 99.85% and *Q*_e_ decreases to 142.64 mg/g. This is possibly because the ML@CS amount was low at the beginning and the number of adsorption sites was less when *C*_0_ was kept at a certain concentration, only a small amount of MO was adsorbed, resulting in low *R* and high *Q*_e._ Upon adding ML@CS, the available adsorption sites increased and the mass transfer power for adsorption became larger. This caused the adsorption of more MO amounts by the active sites on the ML@CS surface, and thus, *R* increased considerably. Meanwhile, the adsorption sites that were not occupied by MO increased gradually. Excessive amounts of ML@CS result in excess adsorption sites or the aggregation and overlap of adsorption sites, which reduce the utilisation rate of adsorption sites and cause a sharp decrease in *Q*_e_ [32]. Considering the adsorption effect and cost, the optimal dosage of ML@CS was determined to be 0.05 g, i.e., 1.00 g/L.

#### 2.3.3. Effect of Solution pH

Acidity, or pH, is one of the important factors affecting adsorption behaviour, which not only affects the surface charge and properties of the adsorbent, but also affects the existence form of MO in solution. At 25 °C, the effect of pH on the adsorption ability of MO by ML@CS was investigated at *C*_0_ of 200 mg/L, W_ML@CS_ of 0.05 g, and adsorption time of 180 min. As shown in Figure 7c, the adsorption performance of ML@CS was considerably influenced by the pH of the solution. In particular, *Q*_e_ and *R* increased first and then decreased with increasing pH. The maximum *Q*_e_ and *R* of 199.52 mg/g and 99.76%, respectively, were obtained at pH 5.0 because pH determined the electrochemical properties of the solution and affected the existing forms of CS and MO in the solution. The change in the proportion of different existing forms can lead to variation in the adsorption mechanism. At pH < 5.85, the Zeta potential shows that the ML@CS surface is positively charged, and the protonations of –NH_2_ and –OH on the ML@CS surface to form amino cation (–NH_3_^+^) and hydroxyl cation (–OH_2_^+^) will also increase the positive charge density on the ML@CS surface [33], these will be conducive to the adsorption of RSO_3_^−^ in wastewater by electrostatic attraction and increase *Q*_e_ and *R*. However, RSO_3_^−^ was not adsorbed at low pH because (1) the weakly alkaline CS was prone to hydrolysis, (2) the enhanced positivity of MO repelled the positive charge on the ML@CS surface and (3) the excess H^+^ in the solution competed with –NH_3_^+^ and –OH_2_^+^ on the ML@CS surface. When pH > 5.85, the Zeta potential shows that the ML@CS surface is negatively charged, meanwhile (1) –NH_2_ on CS is not easily protonated and –OH is easily converted to oxygen anion (–O^−^), which did not facilitate the electrostatic interaction between CS and RSO_3_^−^, and (2) excessive OH^−^ group in the solution resulted in competitive adsorption with RSO_3_^−^ and decreased the electrostatic adsorption capacity of MO on the ML@CS surface, thereby decreasing *Q*_e_ and *R* [34]. In addition, the existing form of MO at various pH affected the adsorption. At pH < 3.1, the red *p*-quinone structure was the dominant form. At pH > 4.4, yellow azo was the main structure. The azo structure was more stable than the quinone structure and was more difficult to be removed. A certain adsorption amount was observed at higher or lower pH due to the formation of hydrogen bonds between the highly electronegative oxygen and nitrogen atoms in the MO molecule and the –OH of ML@CS [35]. Thus, the optimum pH for MO adsorption by ML@CS was deemed to be 5.0.

#### 2.3.4. Effect of Adsorption Temperature and Time

The effects of temperature and adsorption time on MO were studied at *C*_0_, *W*_ML@CS_, and pH of 200 mg/L, 0.05 g, and 5.0, respectively. As shown in Figure 7d, both *Q*_t_ and *R* first increase dramatically and then gradually as the adsorption time increases at different temperatures. After a certain adsorption time, *Q*_t_ and *R* tend to remain unchanged, indicative of chemical adsorption. Moreover, the adsorption process was slow and required considerable time to reach equilibrium. For example, in the first 150 min, the *Q*_t_ of MO on ML@CS increased rapidly at 25 °C with time. After 150–180 min, *Q*_t_ increased gradually until reaching the adsorption equilibrium (~180 min), and *Q*_t_ and *R* remained nearly unchanged because at the initial stage of adsorption, MO molecules and active groups in ML@CS did not achieve optimal interaction and a large number of unoccupied adsorption sites were present on the ML@CS surface [36]. As the adsorption time increased, the concentration of MO near ML@CS decreased and a concentration gradient formed with the surrounding MO solution owing to the gradual adsorption of MO near ML@CS. This facilitated the rapid diffusion of MO in the high-concentration area near the periphery of ML@CS, thereby dramatically increasing *Q*_t_ and *R*. As the adsorption sites and concentration gradient on the ML@CS surface decreased, *Q*_t_ and *R* increased only gradually. When the active sites on the ML@CS surface tended to be saturated, a dynamic balance of adsorption and desorption was achieved and *Q*_t_ remained almost unchanged. As can be seen from Figure 7d, with the increase of temperature, *Q*_t_ decreases at the same time and the time to reach adsorption equilibrium shorten, but the adsorption equilibrium can be reached within 180 min in both cases, indicating that the adsorption of ML@CS on MO is an exothermic process under experimental conditions, and the increase of temperature inhibits the forward adsorption and decreases the adsorption capacity [37]. Therefore, the optimal adsorption time at 25 °C is 180 min.

#### 2.3.5. Evaluation of the Regeneration Performance

**Figure 8:**
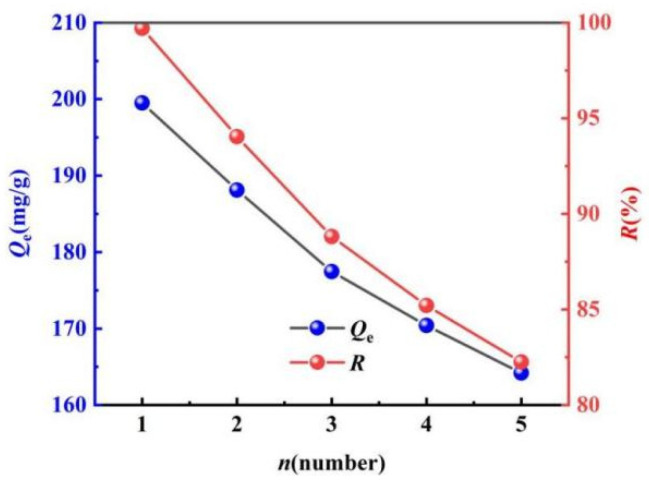
Recycling effect of ML@CS.

Taking both the application performance and economy into consideration, the regeneration performance of ML@CS is shown in Figure 8.

MO can be released from ML@CS by exchanging OH^−^ with the anion MO, thereby realising the regeneration and reuse of ML@CS. Solid–liquid separation of regenerated ML@CS can be easily performed than that of CS under centrifugation owing to the gravity of the loess. As shown in Figure 8, both *Q*_e_ and *R* decreased with the increase of cycle, but *Q*_e_ and *R* decreased from 199.52 mg/g and 99.76% to 164.18 mg/g and 82.25% after five cycles, and *Q*_e_ and *R* decreased by about 7.06 mg/g and 3.50% after each cycle, respectively, and no significant change was observed in appearance and mechanical strength of ML@CS, indicating that ML@CS has good mechanical strength and regeneration performance. The adsorption efficiency decreased after cyclic regeneration because (1) some adsorption sites adsorbed MO too firmly and it could not be effectively desorbed by sodium hydroxide solution and (2) the structure of ML@CS was partially destroyed during desorption, reducing the number of adsorption sites.

### 2.4. Analysis of the Desorption Mechanism

#### 2.4.1. Adsorption Isothermal Analysis

**Figure 9:**
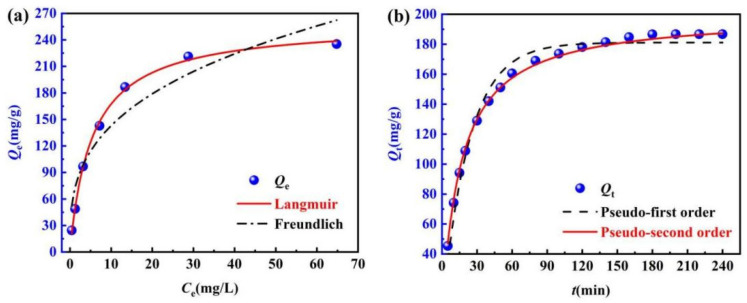
Adsorption curves and fitting graphs of MO by ML@CS. (**a**) Adsorption isotherms. (**b**) Adsorption kinetics.

The adsorption isotherm can describe the relationship between the equilibrium concentration of adsorbent and the adsorption amount, and reveal the interaction between adsorbent and adsorbent [38]. Freundlich and Langmuir models are fitted with Matlab and Origin software to the adsorption data, and the results are shown in Figure 9a and Table 2.

Figure 9a shows that the *Q*_e_ of ML@CS for MO increases with *C*_e_ at a certain temperature. Table 2 shows the adsorption parameter 1/*n* of 0.325 < 0.5 in the Freundlich isotherm model, suggesting adsorption easily occurred [39]. However, the *R*^2^ (0.997) of the Langmuir model was significantly higher than that of the Freundlich model (*R*^2^= 0.897), and *k*_L_ (0.189) and *R*_L_ were in the range of 0–1, indicating that the adsorption behaviour of ML@CS for MO is more suitable to be described using the Langmuir model. It is mainly homogeneous adsorption by single molecular layer, which is conducive to adsorption and there is no interaction between the adsorbed substances [40]. At the adsorption temperature of 25 °C, the *Q*_max_ calculated by the Langmuir equation is 259.76 mg/g, which was obviously higher than the saturated adsorption capacity of traditional adsorbents such as activated carbon (113.63 mg/g), activated carbon nanotubes (149 mg/g), and chitosan nanocomposites (93.84 mg/g) for MO at the same temperature [34,41,42]. These results confirmed the excellent adsorption effect of ML@CS for MO.

#### 2.4.2. Analysis of the Adsorption Kinetics

The pseudo-first-order and pseudo-second-order kinetic models are fitted with Matlab and Origin software to the adsorption data at 25 °C, and the results are shown in Figure 9b and Table 3.

Figure 9b and Table 3 show that the pseudo-first-order and pseudo-second-order kinetic models had high correlation coefficients (*R*^2^ > 0.95). This indicates that the experimental data agreed well with the results of these models and physical and chemical adsorption may occur. However, the correlation coefficient (*R*^2^ = 0.998) of the quasi-second-order kinetic adsorption model was higher than that of the quasi-first-order kinetic model (*R*^2^ = 0.977), and the fitted *Q*_e, cal_ (200.00 m/g) was closer to *Q*_e, exp_ (199.52 mg/g), signifying that the adsorption behaviour of ML@CS for MO can be better explained using the quasi-second-order kinetic adsorption model.

In conclusion, the adsorption capacity of ML@CS for MO was related to the adsorption sites on its surface. The adsorption rate mainly focused on the chemical adsorption with hydrogen bonding and electrostatic attraction between ML@CS and MO [43] and was supplemented by physical adsorption. The fitting data of adsorption kinetics were consistent with the adsorption isotherm data.

#### 2.4.3. Analysis of Adsorption Mechanism

**Figure 10:**
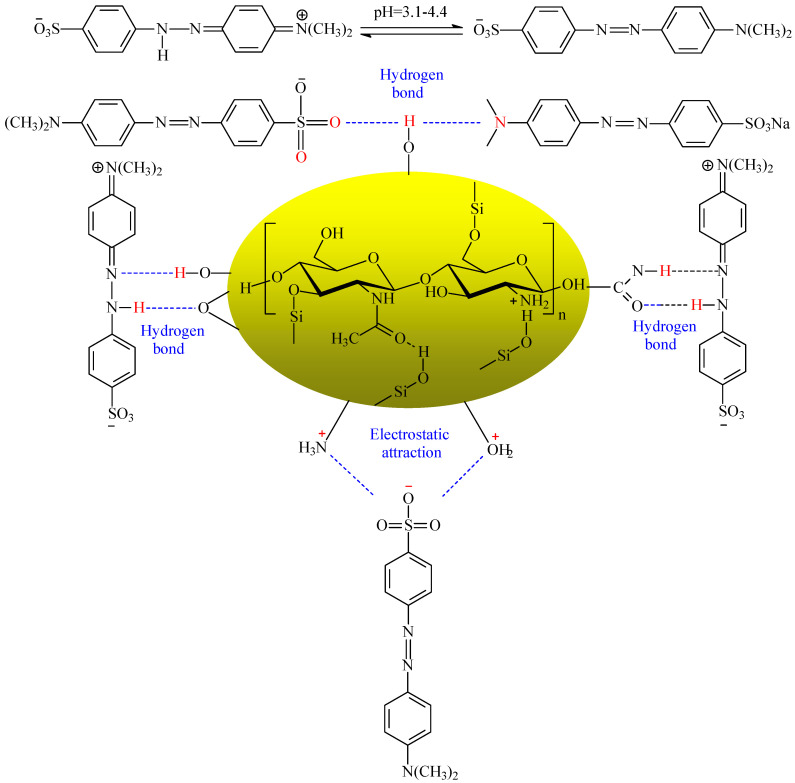
Adsorption mechanism of MO by ML@CS. The yellow oval and the structure in it represent ML@CS, and the surface –OH, –NH_2_, –OH_2_^+^, –NH_3_^+^ and –O– form electrostatic and hydrogen bonds with MO.

The findings related to adsorption conditions, adsorption kinetics and adsorption isotherms of ML@CS for MO showed that ML@CS exhibited a distinctive adsorption performance for MO, and adsorption mainly occurred based on monolayer chemical adsorption, wherein the physical adsorption only played an auxiliary role. This is because (1) MO molecules mainly diffused into the ML@CS pores under zero potential, (2) –NH_3_^+^ and –OH_2_^+^ obtained from the protonation of –OH and –NH_2_ in ML@CS had strong electrostatic attraction with RSO_3_^−^ in MO, and (3) –OH in ML@CS can be adsorbed through van der Waals force or hydrogen bonds with highly electronegative O and N atom in MO molecules [35,44]. Figure 10 shows the adsorption mechanism.

## 3. Materials and Methods

### 3.1. Materials and Instruments

Loess (30 cm deep under the earth’s surface layer) was collected from Zhangtiegou in Balimiao Village, Wenquan Township, Xifeng District, Qingyang City, and the impurities on the surface were removed before use. Chitosan (deacetylation degree ≥ 95%), MO (Analytical purity, Guoyao Group Chemical Reagent Co., Ltd., Beijing, China), acetic acid, hydrochloric acid, sodium hydroxide, and ethanol were analytically pure (Tianjin Tianli Chemical Reagent Co., Ltd., Tianjin, China). Deionised water was used for the experiments.

The experiment used the following instruments: Fourier transform infrared spectrometer (FTIR-8400S, Shimadzu Corporation, Kyoto, Japan), adsorption specific surface area aperture distributor (Tristar II 3020, Mackbrothers LLC, Charlotte, NC, USA), X-ray diffractometer (D/max-RB, Rigaku Holdings Corporation, Kyoto, Japan), scanning electron microscope (S-3000N, Hitachi High-Technologies Corporation, Kyoto, Japan), Zeta potential analyser (JS94H, Zhongchen Digital Technology Equipment Co., Shanghai, China), thermogravimetric analyser (Q-500, TA Instruments, New Castle, DE, USA), precision pH metre (PHS-3C, Lei Magnetic Instrument Factory, Shanghai, China), ultraviolet–visible spectrophotometer (754N, Aoyi Scientific Instrument Co., Shanghai, China), visible spectrophotometer (722S light Technology Co., Shanghai, China), electric centrifuge (80–1, Danrui Experimental Equipment Co., Changzhou, China), electronic balance (AB204-N, Mettler Ledoequipment Co., Zurich, Switzerland), vacuum-drying oven (DZF-6020, Ganyi Instrument Equipment Co., Shanghai, China), water bath thermostatic oscillator (SHZ-88, Peiying Experimental Equipment Co., Suzhou, China), digital thermostatic water bath (HH-1, Jerer Electric Appliance Co., Changzhou, Jiangsu, China), ultrasonic cleaner (410T, Guanyuan Technology Co., Beijing, China), electro-thermostatic blast oven (DHG-9140A, Aoke Environmental Test Equipment Co., Hangzhou, China), and timer-controlled magnetic stirrer (MYP13-2D, Shanghai Qianjun Scientific Instrument Co., Shanghai, China).

### 3.2. Loess Modification

A sample of 100.0 g of natural loess ground through 50-mesh sieve was added to 500 mL of deionised water, stirred, and fully dispersed. After standing the mixed system for 120 min, impurities such as suspended solids in the upper layer and fine sand in the bottom were removed, and the loess mud in the middle layer was taken out. The above operation was repeated twice, and finally the loess mud was centrifuged, dried at 110 °C, and ground through a 100-mesh standard sieve to obtain the pre-treated loess powder. The loess powder was then added to 500 mL of 3 mol/L hydrochloric acid, heated to 100 °C under condensation reflux and stirred at 300 r/min for 120 min. After the reaction, the mixture was cooled to room temperature and filtered, and the filter cake was washed repeatedly with deionised water until the filtrate was neutral (pH = 7). The filter cake was then dried at 110 °C to constant weight, ground and passed through a 100-mesh sieve to obtain modified loess (ML).

### 3.3. Preparation of ML@CS

A total of 2.0 g of CS was added to 100 mL of 5% acetic acid solution and stirred at 70 °C until it was completely dissolved. A small amount of distilled water was added to 10.0 g of the ML. This mixture was dispersed using ultrasonic waves and added to the CS solution. After magnetic stirring at 60 °C for 180 min to fully reaction, the mixture was centrifuged and the solid was collected. The solid was washed repeatedly with deionised water until the filtrate became neutral, and then dried at 80 °C, ground, and passed through a 100-mesh sieve to obtain ML@CS.

### 3.4. Characterisation of the Prepared Composite

The composition and changes of the materials were characterized by FT-IR spectroscopy (the sample was mixed with standard KBr and pressed into tablets; the scanning range was 400~4400 cm^−1^). The surface morphology of the composite and the adhesion of CS on ML surface were observed via SEM. Approximately 5 nm-thick gold layer was coated on the sample, and the voltage was 4.9 kV. The phase and crystal structure of the materials were analysed by XRD method (tube potential of 40 kV, tube current of 40 mA, scanning angle of 20°–80°, scanning speed of, 4°/min). The thermal stability of the material was determined via TGA under an N_2_ atmosphere at 800 °C and 10 °C/min. The Zeta potential of dispersion was also tested.

### 3.5. Plot of the Standard Curve of MO

A series of MO standard solutions with concentrations (*C*) of 2, 4, 6, 8, 10, and 12 mg/L were prepared. The 10 mg/L solution was scanned using an ultraviolet–visible spectrophotometer at 300–600 nm, and λ_max_ of MO was determined as 464 nm. Then, distilled water was used as the reference, and the absorbance (*A*) of the MO standard solutions was measured at λ_max_ using a 722S visible spectrophotometer (the measurement approach of absorbance for the following solution was completely consistent with the aforementioned statement). Taking *A* as the ordinate and *C* as the abscissa, the standard curve of *A* = 0.0714*C* − 0.0027 (*R*^2^ = 0.9993) was plotted.

### 3.6. Adsorption Tests

#### 3.6.1. Investigation of the Adsorption Conditions

A 50 mL volume of MO solution with a known mass concentration was accurately measured and placed in a 150 mL-conical flask. The pH of solution was adjusted to the set value using 0.1 mol/L HCl or NaOH solution and determined using PHS-3C type precision pH meter (the following methods for adjusting pH were the same), to which a certain amount of ML@CS was added. The conical flask was sealed and shaken at 300 r/min for a certain time in a 25 °C thermostatic oscillator, the resultant mixture was then filtered through a 0.22 μm filter membrane, and the absorbance of the filtrate was determined at λ_max_. All the experiments were conducted thrice in parallel, and the mean values were obtained. Finally, the mass concentration of MO solution was analysed using the standard curve, and the adsorption capacity *Q* (mg/g) and removal rate R (%) were calculated according to Equations (1) and (2), respectively, as follows:

(1) $$Q\;=\frac{(C_{0}-C)\times V}{W}$$

(2) $$R=\frac{C_{0}-C}{C_{0}}\times 100\%$$

Here, *C*_0_ and *C* are the mass concentrations of MO in the solution before and after adsorption (mg/L), respectively. *W* is the mass of ML@CS (g) and *V* is the volume of MO solution (L).

The effects of initial concentration of MO, solution pH, ML@CS dosage, adsorption temperature, and time on the adsorption effect of MO were respectively investigated using *Q* and *R* as indexes.

#### 3.6.2. Establishment of the Adsorption Kinetic Model

A 50 mL volume of MO solution with an initial concentration of 200 mg/L was accurately measured, and pH was adjusted to 5.0. After adding 0.05 g ML@CS, the flask was sealed and shaken at a constant temperature of 25 °C (300 r/min), during which samples were taken every 20–240 min. After the sample was filtered through a 0.22 μm filter membrane, and the absorbance of the filtrate was measured at λ_max_. All the tests were performed thrice in parallel, and the average value was acquired. *Q*_t_ and *Q*_e_ were calculated using Equation (1), and the adsorption kinetics were fitted using the pseudo-first-order kinetic model (Equation (3)) and pseudo-second-order kinetic model (Equation (4)).

ln(*Q*_e_ − *Q*_t_) = ln*Q*_e_ − *k*_f_*t* (3)

(4) $$\frac{t}{Q_{t}}=\frac{1}{k_{s}{Q_{e}}^{2}}+\frac{t}{Q_{e}}$$

Here, *Q*_t_ and *Q*_e_ are the adsorption capacities (mg/g) at time *t* and equilibrium, respectively, *t* is the adsorption time (min), *k*_f_ is the pseudo-first-order adsorption rate constant (min^−1^), and *k*_s_ is the pseudo-second-order adsorption rate constant (g/mg·min).

#### 3.6.3. Establishment of the Isothermal Adsorption Model

A 50 mL volume of MO solution with an initial concentration of 25–300 mg/L was accurately measured and its pH was adjusted to 5.0, followed by the addition of 0.05 g ML@CS. The flask was then sealed and the solution was shaken (300 r/min) at a constant temperature of 25 °C for 180 min in a thermostatic oscillator. After filtration through a 0.22 μm filter membrane, the absorbance of the filtrate was measured at λ_max_. All the tests were conducted thrice in parallel and the average value was regarded as the final experimental value. *Q*_e_ was determined using Equation (1). The isothermal adsorption curve was fitted using Langmuir (Equations (5)) and Freundlich (Equation (6)) models, and the separation factor (*R*_L_) was calculated using Equation (7) [45].

(5) $$\frac{C_{e}}{Q_{e}}=\frac{1}{k_{L}Q_{\max}}+\frac{C_{e}}{Q_{\max}}$$

(6) $$\ln Q_{e}=\ln k_{F}+\frac{\ln C_{e}}{n}$$

*R*_L_ = (1 + *k*_L_*C*_0_)^−1^ (7)

where *C*_0_ and *C*_e_ denote the mass concentrations (mg/L) of MO in the solution before adsorption and equilibrium, respectively. *Q*_e_ denotes the adsorption capacity (mg/g) at adsorption equilibrium, *Q*_max_ denotes the maximum adsorption capacity (mg/g) from the point of theory, *k*_L_ denotes the Langmuir isothermal adsorption constant (L/mg), *k*_F_ denotes the Freundlich isothermal adsorption constant (mg^1−1/n^·L^1/n^/g), *n* is the Freundlich adsorption coefficient (dimensionless), and *R*_L_ denotes the separation factor, dimensionless.

#### 3.6.4. Evaluation of Regeneration Performance

Regeneration was an important index for evaluating the adsorption of materials. A superior regeneration performance is conducive to saving resources and reducing production costs. ML@CS saturated with adsorbed MO was added to a 0.5 mol/L NaOH solution, which was shaken at a constant temperature of 25 °C (150 r/min) for 2 h. Then, the product was centrifuged and the resultant solid was washed with ethanol and deionised water 3 to 5 times. The solid was dried at 80 °C to obtain the regenerated ML@CS. Under the optimised adsorption conditions, the regenerated ML@CS was used to adsorb MO until reaching the adsorption equilibrium. Then, the corresponding *Q*_e_ and *R* values were calculated.

## 4. Conclusions

(1) ML@CS adsorption materials were prepared via the solution blending method using loess and chitosan as the main raw materials. ML provided support for CS crosslinking network, which is conducive to improving the stability of CS in an acidic environment, its adsorption, and its mechanical and regeneration properties [46].

(2) ML@CS had a fast adsorption rate, large adsorption capacity, and easy sedimentation and separation for MO. When the initial concentration of MO was 200 mg/L, pH was 5.0, ML@CS dosage was 1.00 g/L at room temperature, and the adsorption time was 180 min, *R* and *Q*_e_ reached 99.75% and 199.52 mg/g, respectively, and the theoretical *Q*_max_ was 259.76 mg/g. The residual concentration of MO in wastewater after the adsorption treatment was <0.5000 mg/L, which meets the discharge standard.

(3) The adsorption behaviour of MO on ML@CS conformed to the pseudo-second- order kinetic model and Langmuir isothermal adsorption, indicating a spontaneous and exothermic monolayer chemical adsorption process.

(4) The adsorption mechanism of MO on ML@CS was as follows: MO molecules diffused into the pore structure of ML@CS, and an electrostatic attraction occurred between RSO_3_^−^ in MO molecules and the positive charge on the ML@CS surface. Moreover, -OH on ML@CS formed hydrogen bonds with O and N in MO molecules or through van der Waals Force to finish the adsorption.

(5) ML@CS can be fabricated using abundant raw materials and a simple preparation process. It is economical, has good recycling performance, and does not cause secondary pollution. ML@CS can be used as an anionic dye adsorption material owing to its environmental protection and regeneration properties. These advantages make this a potential application prospect in the field of printing and dyeing wastewater treatment.

## Figures and Tables

**Table 1:** BET parameters of ML and ML@CS.

Materials	Specific Surface Area/(m^2^/g)	Mean Pore Volume/(cm^3^/g)	Mean Aperture/ nm
ML	10.63	0.228	2.43
ML@CS	59.34	0.207	2.25

**Table 2:** Fitting parameters of adsorption isotherm models of MO by ML@CS.

*T*/K	Freundlich			Langmuir		
	*k*_F_/(mg^1−1/*n*^·L^1/*n*^/g)	1/*n*	*R* ^2^	*Q*_max_/(mg/g)	*k*_L_/(L/mg)	*R* ^2^
298	67.61	0.325	0.897	259.76	0.189	0.997

**Table 3:** Fitting parameters of the adsorption kinetics models of MO by ML@CS.

*C*_0_/ (mg/L)	*Q*_e, exp_/ (mg/g)	Pseudo-First-Order			Pseudo-Second-Order		
		*k*_f_/min^−1^	*Q*_e_/(mg/g)	*R* ^2^	*k*_s_/(g/mg·min)	*Q*_e_/(mg/g)	*R* ^2^
200	199.52	0.044	181.13	0.977	0.0003	200.00	0.998

## Data Availability

No new data were created or analyzed in this study. Data sharing is not applicable to this article.
